# Scalable, High‐Density Expansion of Human Mesenchymal Stem Cells on Microcarriers Using the Bach Impeller in Stirred‐Tank Reactors

**DOI:** 10.1002/bit.70025

**Published:** 2025-07-17

**Authors:** Tom A. Wyrobnik, Laia Miranda, Alan Lam, Steve Oh, Andrea Ducci, Martina Micheletti

**Affiliations:** ^1^ Department of Biochemical Engineering UCL London UK; ^2^ Bioprocessing Technology Institute, A*STAR Singapore Singapore; ^3^ Department of Mechanical Engineering UCL London UK

**Keywords:** bioprocessing, engineering characterization, mesenchymal stem cells, microcarriers, stirred‐tank reactors

## Abstract

This paper describes the results of process developmental experiments to achieve higher cell densities in the manufacturing of hMSCs using the novel Bach impeller in a stirred‐tank bioreactor. Engineering experiments have previously shown that the Bach impeller represents an efficient mixing device that suspends particles in fluids at low power inputs. To assess the impeller during biological experiments, the growth performance of Wharton Jelly (WJ)‐hMSCs in a 1 L STR equipped with the Bach impeller was evaluated at a variety of culture conditions. The cells attached to Cytodex 1 microcarriers at a concentration of 5.6 g/L and were cultured for 5–7 days. The growth phase was carried out at varying impeller speeds N = 75, 115, and 150 rpm. Cell growth was additionally evaluated at a microcarrier concentration of 11.2 g/L Cytodex 1. Here, a maximum cell density of up to 1.7 × 10^6^ cells/mL and cell viability > 90% was achieved within 5 culture days, which is amongst the highest cell densities ever attained for a hMSC batch culture. Critical cell quality attributes of the WJ‐hMSCs were assessed upon completion of the growth phase, that is, FACS to identify stem cell surface markers, tri‐lineage differentiation, and capacity of the cells to form colonies. In addition, informed by the previously described engineering characterization, the 1 L process at N = 75 rpm was scaled up to the 5 L scale, where WJ‐hMSCs were again confirmed to have retained the relevant cell quality attributes. The reported findings are important to determine the design space to which scale‐ups to even larger tank sizes can adhere.

**Abbreviations**
BSA: bovine serum albumin
ccm: cubic centimeters per minute
CFU‐F: colony‐forming unit fibroblasts
DAPI: 4′,6‐diamidino‐2‐phenylindole
DO: dissolved oxygen
FACS: fluorescence‐activated cell sorting
hMSC: human mesenchymal stem cell
MC: microcarrier
OUR: oxygen uptake rate
PBS: phosphate‐buffered saline
PFA: paraformaldehyde
P/S: penicillin‐streptomycin
*R*²: coefficient of determination
SD: standard deviation
STR: stirred‐tank reactor
WJ: Wharton's Jelly
αMEM: alpha‐minimum essential medium

## 1 Introduction

Human mesenchymal stem cells (hMSCs) possess significant therapeutic potential in regenerative medicine, owing to their immunomodulatory properties and capacity to differentiate into multiple cell lineages. Clinical applications, particularly in the context of allogeneic therapies, often necessitate the production of large cell doses, typically ranging from 10⁶ to 10⁹ cells per patient, depending on the indication (Chen et al. 2013). Although conventional two‐dimensional (2D) culture systems can achieve adequate cell densities, they present major limitations for large‐scale manufacturing due to restricted surface area, labor‐intensive handling, and limited process control. These limitations collectively motivate the adoption of scalable bioreactor‐based systems (Alici and Blomberg 2010; Heathman et al. 2015). Over the past decade, different bioreactor platforms have been explored for hMSC expansion, such as hollow fibers (Cheatham et al. 2019), vertical‐wheels (Yuan et al. 2018), and stirred‐tank reactors (STRs) (Goh et al. 2013; Rafiq et al. 2013; Tan et al. 2016). Research efforts have predominantly focused on reactors using impellers, given their proven track record and widespread use in the biologics and biotherapeutics industries (Kehoe et al. 2010). Here, valuable advances have been made in culturing hMSCs with regard to the culture medium (e.g., feeding optimization, xeno‐free culture medium development), microcarrier (MC) engineering (e.g., biodegradable/dissolvable MCs, surface functionalization), and general processing steps such as seeding, expansion, harvesting, and subsequent cryopreservation (Dos Santos et al. 2010; Eibes et al. 2010; Lam et al. 2017; Tan et al. 2015).

Rigorous engineering characterization has become increasingly important in the cell therapy industry, as the product (i.e., the cells themselves) is highly sensitive to the flow environment within the bioreactor (Tsai et al. 2020; Zhao et al. 2007). Fluid dynamics studies of stirred reactors, which examine variations in impeller and vessel design, have traditionally been conducted using laser‐based techniques and/or computational fluid dynamics simulations for chemical engineering applications (de Lamotte et al. 2018; Ducci and Yianneskis 2006), and more recently, for bioprocess development (Delbridge et al. 2023a, 2023b; Samaras et al. 2019; Wyrobnik et al. 2022). Such studies serve to support and inform bioprocesses in three key ways: (i) by enabling improvements in process outcomes through the identification of quantifiable parameters; (ii) by optimizing impeller designs and mixing devices to increase the efficiency of specific characteristics, such as reducing power consumption or meeting specific process requirements; and (iii) by providing critical insights for scaling up to larger reactors, through the identification of limiting parameters that can be quantified and controlled.

In earlier investigations, we reported the engineering characterization of the Bach impeller (Wyrobnik et al. 2022). This impeller demonstrated efficient particle suspension at low power inputs and reduced shear environments, making it particularly suitable for sensitive cell types such as hMSCs. The initial characterization was conducted in the absence of internal components, such as probes and addition lines. However, prior studies have demonstrated that such internal components can significantly influence fluid dynamics and power consumption within STRs, often mimicking the hydrodynamic effects of baffles even in nominally unbaffled configurations (Charalambidou et al. 2024). Building upon this foundational work, the present study extends the analysis by assessing the impact of internal components in conjunction with the Bach impeller. Furthermore, we report, for the first time, oxygen transfer data associated with this impeller. Engineering characterization is integrated with biological experimentation, focusing on the expansion of Wharton's Jelly‐derived human mesenchymal stem cells (WJ‐hMSCs) on MCs in stirred‐tank bioreactors. We evaluate the impact of different impeller speeds on cell growth, viability, and quality attributes at both the 1 and 5 L scales. Additionally, we investigate how the previously characterized hydrodynamic parameters translate across scales to inform a rational scale‐up strategy. This study aims to define a scalable and robust process for high‐density WJ‐hMSC cultivation, combining engineering and biological evaluations to support future clinical manufacturing applications.

## 2 Methodology

### 2.1 Engineering Characterization

#### 2.1.1 Bioreactor and Impeller Configuration

**Figure 1:**
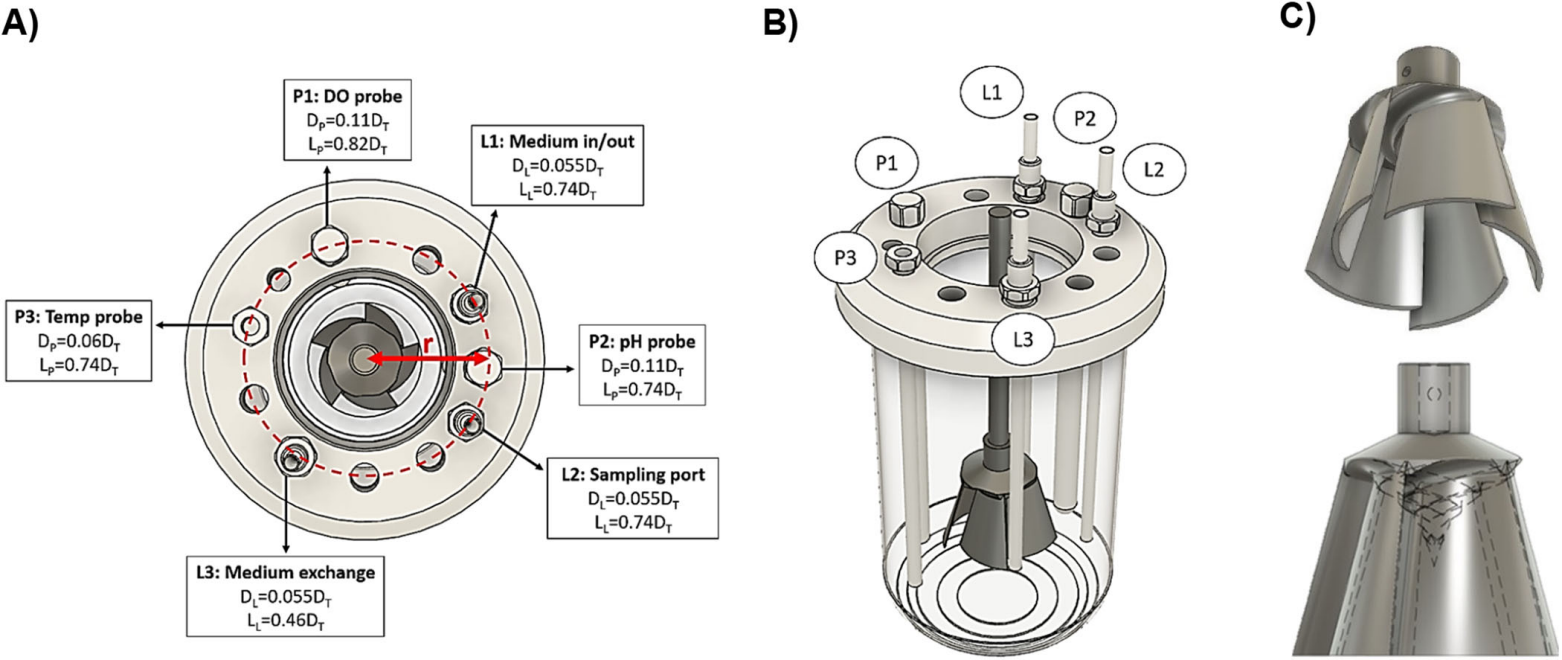
Configuration and detailed views of the UniVessel 1 L bioreactor and Bach impeller. (A) Top and (B) isometric view of the Univessel 1 L bioreactor. The bioreactor is equipped with three probes (temperature, pH, and dissolved oxygen) and three fluid exchange lines. (C) Bach impeller. Adapted from Wyrobnik et al. (2022) and Wyrobnik (2023). D_L_, line diameter; D_P_, probe diameter; L, line; L_L_, line length; L_P_, probe length; P, probe.

A custom‐designed acrylic bioreactor vessel was employed to replicate the key dimensions of the commercially available UniVessel 1 L glass bioreacteor from Sartorius (tank height: *H* = 180 mm; tank diameter: T = 110 mm). The vessel featured a dished bottom, cylindrical body, and was free of internal baffles. Its top lid included a large central port for the impeller shaft insertion, along with 11 smaller ports arranged in a circular pattern (radius: r = 45 mm) to accommodate additional components. Six internal elements, three probes and three lines (hereinafter referred to as “probes”), were submerged within a 1 L liquid volume, matching the dimensions of components used in the UniVessel reactor (Figure 1A,B). These included a temperature probe (Sartorius), a dissolved oxygen probe (Hamilton, OxyFerm FDA 160, Cat.: 237455), a pH probe (Hamilton, EasyFerm Plus PHI K8 160, Cat.: 238633‐1523), and three metal dip tubes (Sartorius) for fluid exchange and sampling. Mixing was achieved using a 3D‐printed Bach impeller (D/T = 0.52, C = 0.33T) (Cell Motions, Hamilton, Canada; Figure 1C). The impeller was powered by a centrally mounted Allen Bradley motor connected to an Ultra3000 Servo drive and controlled via Ultraware software.

#### 2.1.2 Power Input Measurement

The ungassed power input of the Bach impeller across various rotational speeds was measured following the protocol by Wyrobnik et al. (2022). In this setup, the bioreactor vessel was mounted on an air‐bearing system, with a digital gauge (DFG55‐10, Omega Engineering) secured on a fixed scaffold around the vessel to record the force exerted by the rotating impeller. Data were collected at a rate of 10 Hz and averaged over a 60‐s interval, with each condition tested in triplicate (*n* = 3). All measurements were conducted at room temperature using either deionized water or deionized water‐glycerol mixtures (purity > 99%, Thermo Fisher Scientific, UK). Dynamic viscosities were determined using a Kinexus lab+ rheometer (Netzsch‐Gerätebau GmbH).

#### 2.1.3 Particle Image Velocimetry (PIV)

PIV was used to capture flow characteristics around the Bach using a 2D setup that included a green diode laser, cylindrical lens, mirror, and an intensified camera (Dantec Dynamics A/S). The laser sheet, which could be oriented vertically or horizontally, illuminated neutrally buoyant, rhodamine‐coated particles in the fluid, while a potassium iodide–glycerol mixture ensured refractive index matching with the acrylic components to minimize optical distortions. The recorded images were processed via multi‐pass adaptive correlation analysis in PIVlab to extract both instantaneous and phase‐averaged velocity fields and derive parameters such as shear stress. Maximum shear stress, indicative of the local forces within the flow, was calculated by multiplying the maximum shear rate (obtained from the strain rate tensor) with the fluid's dynamic viscosity. Please refer to Wyrobnik et al. (2024) for more information.

#### 2.1.4 Oxygen Mass Transfer Rate

The volumetric oxygen mass transfer coefficient ($k_{L}a$) was determined via the gassing‐out method. The bioreactor was configured with all relevant metal dip tubes and probes typically used during cell culture experiments. The vessel was filled with 1 L of 1× phosphate‐buffered saline (PBS; 1st Base) solution maintained at 37°C. The $k_{L}a$ was assessed across a range of impeller speeds (N = 50–300 rpm, in 50 rpm increments) with an impeller clearance C = 0.5T. After the working solution reached 37°C, the DO was depleted to 0% through N_2_ sparging. Once quasi‐stationary fluid flow was reached, the air supply into the headspace of the vessel was set to the desired aeration rate (100, 300, or 500 cm^3^/min). Data acquisition was completed when the saturated oxygen concentration exceeded 80%. A total of *n* = 3 repetitions were performed per experimental condition. The average *k*_*L*_ *a* value was used to derive the oxygen transfer rate (OTR) according to Equation 1:

(1) $$\text{OTR}={\text{k}}_{\text{L}}\text{a}\cdot ({\text{C}}^{*}-{\text{C}}_{\text{DO}}),$$

where $C^{*}$ is the oxygen concentration (mg/L) when the liquid is fully saturated with air, and $C_{\mathrm{DO}}$ is the oxygen concentration in the liquid (mg/L). At 37°C, air‐saturated PBS was found to contain an oxygen concentration of 0.214 μmol/mL or 6.85 mg O_2_/L (von Heimburg et al. 2005). A default DO = 30% was assumed for the liquid (2.05 mg O_2_/L), reflecting the target oxygen level typically maintained in the bioreactor during cell culture experiments.

### 2.2 Cell Culture Methodologies

#### 2.2.1 Monolayer Expansion of WJ‐hMSCs

WJ‐hMSCs, sourced from PromoCell (Germany), were cultured in α‐minimum essential medium (αMEM; Gibco) supplemented with 10% fetal bovine serum (HyClone) and 1% penicillin‐streptomycin (P/S; Gibco). All WJ‐hMSCs used in this study were derived from a single donor lot, where *n* = 3 refers to technical replicates using this single donor‐derived cell line. Cells were initially seeded in T‐flasks at a density of 3400–5700 cells/cm² and maintained at 37°C with 5% CO₂. The culture medium was completely exchanged every 48–72 h, and cells were passaged before reaching 80%–90% confluence. Cells were rinsed with 1× PBS and detached with a 0.25% trypsin/EDTA solution (Gibco) at 37°C for 3 min. Trypsin activity was neutralized by adding fresh αMEM in a volume 1–2 times that of the trypsin solution. After centrifugation at 340*g* for 3 min, the cell pellet was resuspended in culture medium. For bioreactor seeding and spinner flask controls, WJ‐hMSCs were expanded in HYPERFlask cell culture vessels (Corning), each providing a growth surface area of 1720 cm², equivalent to 10 T175 flasks, across its 10 layers. Cells were harvested from the HYPERFlasks using standard procedures involving 0.25% trypsin‐EDTA.

#### 2.2.2 Expansion of WJ‐hMSCs in Spinner Flasks

Single‐use 125 mL spinner flasks (Corning) containing Cytodex 1 MCs in 30 mL of αMEM were seeded at a density of 4000 cells/cm². The culture was initially agitated at 25 rpm for 24 h, after which 20 mL of additional αMEM was added and the agitation rate was increased to 30 rpm. The culture was maintained for up to 7 days, with 50% of the medium replaced with fresh αMEM every 48 h. When the cell concentration exceeded 5 × 10^5^ cells/mL, daily medium exchanges (50% of culture volume) were performed. MCs were allowed to settle for 5–10 min before the careful aspiration of the culture medium. Three daily samples were collected from the spinner flask into microtubes for cell counting, imaging, and metabolite analysis. During sampling, the flask was gently swirled in a circular motion to ensure uniform distribution of cells and MCs throughout the suspension.

**Figure 2:**
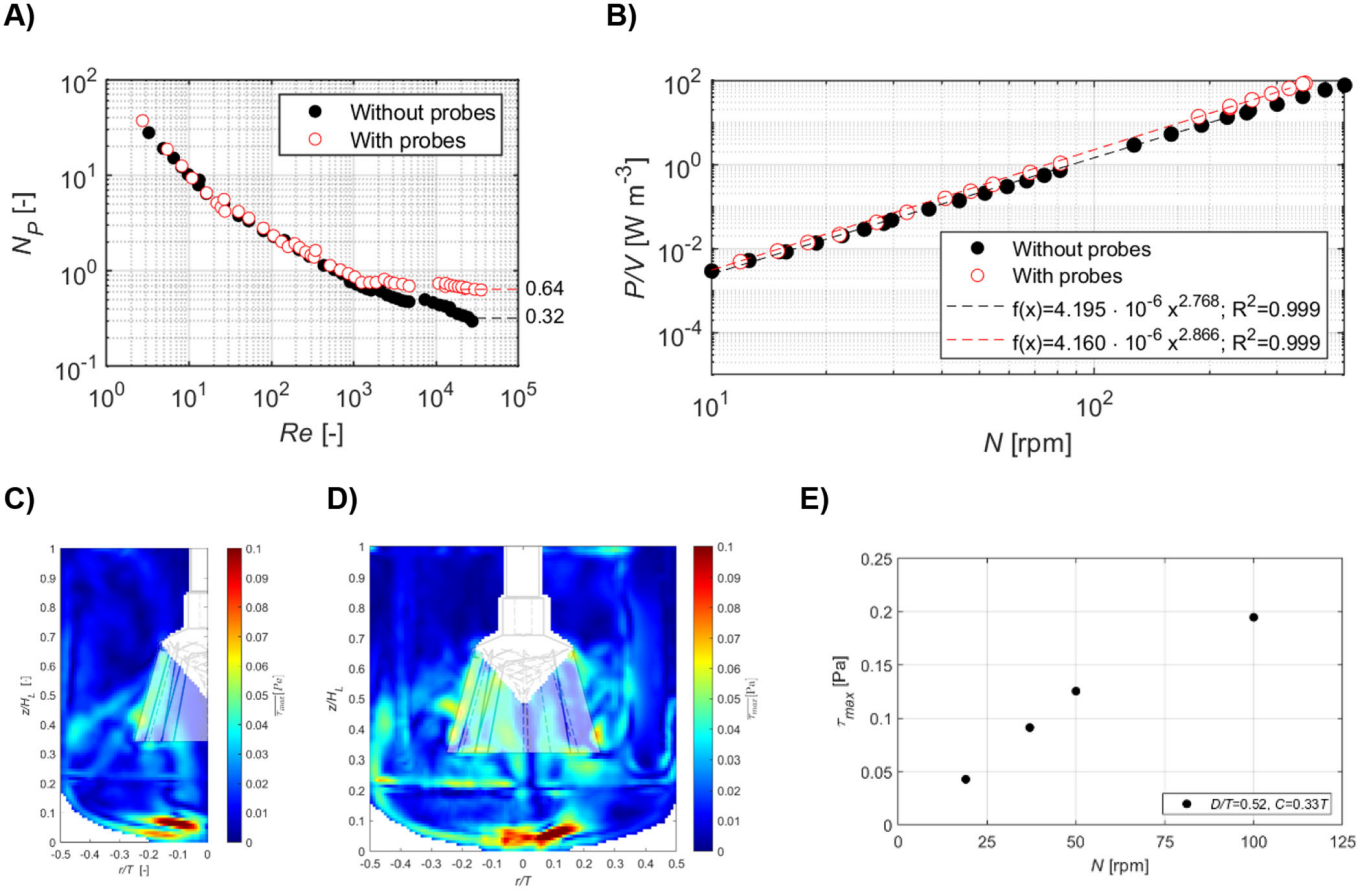
Power characteristics of the Bach impeller with and without submerged probes and lines: (A) Power number ($N_{P}$) curve of the Bach impeller (D/T = 0.52, C = 0.33 T) plotted against Reynolds number (Re), comparing configurations without submerged probes with six submerged culture internals (three probes and three lines). (B) Volumetric power input (P/V) as a function of impeller speed (N) with and without internals. Ensemble‐averaged shear stresses, (C) without or (D) with probes, at Re = 2900 (N = 50 rpm). (E) Maximum shear stresses ($\tau_{\max}$) as a function of N. Data represents the mean (*n* = 3). *C*, impeller off‐bottom clearance; D, impeller diameter; T, vessel tank.

Spinner flasks were selected as a reference system in this study due to their widespread use for MC‐based 3D cell culture (Fernandes et al. 2009; Yuan et al. 2014). This choice is also supported by their mode of agitation. Unlike the static environment of 2D T‐flasks, spinner flasks rely on a stirring impeller similar to that used in small‐scale STRs. For the same 125 mL spinner flask system, Ghasemian et al. (2020) reported peak and mean shear stresses of approximately 0.07 and 0.015 Pa, respectively, at 40 rpm and a 50 mL working volume. Jeske et al. (2021) observed a maximum shear stress of around 0.20 Pa at 30 rpm with 60 mL, while Cantarero‐Rivera et al. (2024) measured a peak of 0.175 Pa and a mean of 0.002 Pa at 30 rpm in 100 mL. Based on the operational conditions used in this study (30 rpm, 30–50 mL), the estimated mean and peak shear stresses are approximately 0.015 and 0.18 Pa, respectively. These values fall within the hydrodynamic range observed in the 1 L STR system used here (see Figure 2).

#### 2.2.3 Bioreactor Cell Culture

WJ‐hMSCs were cultured at two different bioreactor scales: 1 and 5 L. Both the 1 L Univessel Glass bioreactor (Biostat B‐DCU, Sartorius) (T = 110 mm) and 5 L Univessel Glass bioreactor (Biostat B‐DCU, Sartorius) (T = 160 mm) were equipped with a Bach impeller (D/T = 0.52) positioned at an off‐bottom clearance of C = 0.5 T. These configurations were unbaffled and included the previously described internal components (Section 2.1.1). At the 5 L scale, probes and metal dip tubes were proportionally larger. Before use, the bioreactor vessels were siliconized with SigmaCote (ca. 2–4 mL). The seeding density was standardized across all bioreactor cultures at 4000 cells/cm² (equivalent to ∼5 cells/bead). Cytodex 1 MCs were prepared following the manufacturer's guidelines, which included washing with 1× PBS, autoclaving at 121°C for 20 min, and rinsing in αMEM. Working volumes (V_W_) were either 1 or 5 L, depending on the bioreactor scale. Operational conditions remained consistent across both scales: the temperature was set to 37°C, DO was maintained at 30% via overhead aeration, and pH was kept at 7.4 through CO_2_ gassing. The efficiency of cell attachment to MCs was determined by collecting samples at multiple time points post‐seeding. The percentage of attached cells ($A_{\%}$) was calculated as:

(2) $${\text{A}}_{\%}=\frac{{\text{Cx}}_{\text{Susp}}}{{\text{Cx}}_{\text{tot}}}∙100,$$

where ${\mathrm{Cx}}_{\mathrm{Susp}}$ is the viable cell number in suspension and ${\mathrm{Cx}}_{\mathrm{tot}}$ is the total viable cell number (both in suspension and attached to MCs).

Daily samples were collected aseptically in triplicate for cell counting, imaging, and metabolite analysis. Medium exchange was performed with 50% renewal every other day while cell densities were below 5 × 10^5^ cells/mL; once this threshold was exceeded, daily medium exchanges (50% of culture volume) were initiated.

On the final day of culture, 3 × 10 mL were sampled into 50 mL Falcon tubes. The MCs were washed two to three times with 1× PBS, followed by the addition of pre‐warmed 0.25% Trypsin/EDTA solution at four times the volume of the MC suspension. The tube was agitated on a vertical SF1 flask shaker (Stuart) at 300–500 rpm for 3 min at room temperature. If sufficient cell detachment was not achieved, additional shaking was performed, with a maximum total incubation time of 12 min. Once detachment was confirmed, the MC suspension was filtered through 40–70 μm cell strainers (Greiner) into a fresh Falcon tube to separate single cells from MCs. Fresh αMEM, equivalent to 1–2 times the volume of the Trypsin/EDTA solution, was used to neutralize enzymatic activity and wash the filter. The harvested cells were quantified and allocated for subsequent quality control assays. For long‐term storage, cells were cryopreserved in CryoStor CS10 (StemCell Technologies) at −80°C for 24 h before being transferred to liquid nitrogen tanks.

#### 2.2.4 Analytical Techniques and Characterization of Expanded Cells

##### 2.2.4.1 Counting and Population Doubling Time

Cell density was measured using a NucleoCounter NC‐3000 cell counter (Chemometec). Three samples of cells were taken from the single‐cell suspension and placed into separate microtubes. Viable, dead, and total cell counts were determined by staining with Acridine Orange (Thermo Fisher Scientific) and DAPI solution (Thermo Fisher Scientific). Cell viability was automatically calculated as the ratio of live cells to total cells. The fold increase (FI) of the culture was calculated by dividing the final cell count (${\mathrm{Cx}}_{t}$) by the initial cell count (${\mathrm{Cx}}_{0}$):

(3) $$\text{FI}=\frac{{\text{Cx}}_{\text{t}}}{{\text{Cx}}_{0}}.$$

The time required for cells to undergo one population doubling (t_d_) was determined to compare the expansion kinetics of cells grown on MCs with those cultured in standard 2D environments. Cells were thawed and seeded into T‐flasks at a consistent density and expanded over 3–4 passages. Cell counts were used to calculate t_d_ using the following equations:

(4) $${\text{t}}_{\text{d}}=\frac{\ln\left(2\right)}{\mu },$$

(5) $$\mu =\frac{\left(\ln\left(\frac{{\mathrm{Cx}}_{t}}{{\mathrm{Cx}}_{0}}\right)\right)}{∆t},$$

where *μ* represents the specific growth rate (h^−1^) and Δ*t* is the time interval between passages (s).

##### 2.2.4.2 Colony Forming Unit (CFU) Assay

CFU fibroblasts (CFU‐F) assays were conducted by seeding 100, 250, or 500 cells onto six‐well tissue culture plates (Corning) with 4 mL of αMEM. Complete medium exchanges were performed on Days 7 and 10 of incubation. At 14 days post‐seeding, colonies were visualized by crystal violet staining (Sigma‐Aldrich). The staining process began with aspirating the culture medium, followed by a single wash with 1× PBS. Each well was then treated with 3 mL of a 0.5% crystal violet solution and incubated for 30 min at room temperature. After staining, the wells were rinsed three times with 5 mL of 1× PBS. The CFU‐F efficiency ($E_{{\mathrm{CFU}}_{\%}}$) was calculated by dividing the number of colonies counted by the initial number of cells seeded (Equation 6).

(6) $${\text{E}}_{{\text{CFU}}_{\%}}=\left(\frac{\text{Number of counted colonies}}{\text{Number of seeded cells}}\right)∙100.$$

##### 2.2.4.3 Tri‐Lineage Cell Differentiation

Cells harvested from both bioreactor and spinner flask cultures were thawed and cultured for an additional passage to assess their differentiation potential. Differentiation towards adipogenic, osteogenic, and chondrogenic lineages was induced by incubating the cells in respective differentiation media (Sartorius) according to the manufacturer's protocols. After induction, cells were washed with PBS and fixed with 4% paraformaldehyde (PFA; Affymetrix). For differentiation analysis, cells were stained with either Oil Red O to visualize lipid droplets, Alizarin Red S to detect calcium deposition, or Alcian Blue to reveal glycosaminoglycan deposition.

##### 2.2.4.4 Flow Cytometry

Viable WJ‐hMSCs, harvested from both monolayer and MC cultures, were fixed with 4% PFA for 20 min at room temperature and subsequently stored at 4°C for up to 1 month in a 1% (w/v) bovine serum albumin/PBS solution. Surface marker expression was analyzed using flow cytometry on a NovoCyte 2000 instrument (Agilent Technologies) to evaluate the presence of CD34, CD45, CD73, CD90, CD105, and HLA‐DR markers (Biolegend). For each sample, approximately 10,000 events were recorded, and the data were processed using NovoExpress software.

##### 2.2.4.5 Metabolite Analysis

Supernatant from daily bioreactor samples was centrifuged at 20,238*g* for 30 s and frozen at −20°C. On days when medium exchanges were conducted, samples were collected in triplicate both before and after the exchange. Before analysis, the samples were thawed at room temperature and briefly vortexed, then analyzed using a Bioprofile 100 Plus (NOVA) instrument to measure concentrations of glucose, glutamine, lactate, and ammonia.

## 3 Results

### 3.1 Baseline Performance of WJ‐hMSCs Cultured on 2D

WJ‐hMSCs were cultured on a planar surface (T175 flasks) to obtain reference values for critical performance metrics (Figure S1). Key parameters evaluated included population doubling time, CFU efficiency, and tri‐lineage differentiation potential. Population doubling times were measured across multiple passages and following cryopreservation to assess proliferative capacity (Figure S1A). Colony‐forming efficiency was determined by quantifying colony formation from seeded cells at different passages (Figure S1B). Cells were confirmed to express canonical MSC markers (CD73, CD90, CD105) and low expression of hematopoietic and immunogenic markers (CD34, CD45, HLA‐DR) (Figure S1C). The multipotency of the cultured WJ‐hMSCs was validated through adipogenic, chondrogenic, and osteogenic differentiation assays (Figure S1D–F). These data establish the functional baseline of the cell population before bioreactor‐based expansion.

### 3.2 Engineering Characterization of the 1 L STR

The initial cell characterization presented in Section 3.1 provided a baseline for comparing MC bioreactor cultures. Before conducting cell experiments at 1 L scale, a detailed engineering characterization was performed to identify suitable operating conditions that would support the cell expansion; thus reducing the number of final experiments at this scale.

Power measurements were performed across different impeller speeds to evaluate the hydrodynamic environment created by the Bach impeller in a 1 L STR. Both configurations, with and without submerged probes and lines simulating standard bioprocess setups, were tested. The complete power number ($N_{P}$) curve for the Bach impeller was plotted over a Reynolds number (Re) range from approximately 1 to over 2 × 10⁴ (Figure 2A). The introduction of probes did not significantly affect $N_{P}$ at lower Re values (< 10³); however, at Re > 10³, divergence in $N_{P}$ was observed. Specifically, $N_{P}$ without probes continued to decrease, consistent with unbaffled vessel behavior, while $N_{P}$ with probes began to stabilize. At Re > 10⁴ (N > 100 rpm for water‐like fluids), $N_{P}$ for the vessel with probes remained constant, suggesting a transition to turbulent flow, a phenomenon commonly associated with the presence of baffles (Ducci and Yianneskis 2006). Recent studies have shown that internal probes can mimic the baffling effect and increase the power consumption (Charalambidou et al. 2024).

In the configuration with submerged probes, the power number stabilized at $N_{P}$ = 0.64, double the value observed without probes ($N_{P}$ = 0.32). A strong correlation between volumetric power input (P/V) and impeller speed was developed for the vessel with submerged probes, yielding a coefficient of determination (R²) greater than 0.99 (see Figure 2B). The presence of probes was observed to increase P/V across all tested impeller speeds, indicating that additional energy was required due to probe interference. High P/V values have been linked to increased shear stress, which can adversely affect cell viability and proliferation (Hewitt et al. 2011; Nienow et al. 2016; Nienow et al. 2016). In fact, increased energy dissipation may alter metabolic activity; cells might allocate resources toward stress responses rather than growth.

The shear profile corresponding to a Re = 2900, achieved at an impeller speed of 50 rpm, is shown in Figure 2C,D, representing configurations without and with probes, respectively. Thresholds for shear stress known to influence hMSCs, particularly with respect to gene and protein expression, are typically reported in the range of 0.25 to 2 Pa (Wyrobnik et al. 2020). At 100 rpm, the bioreactor exhibited similar shear stress distributions with and without the presence of probes, suggesting minimal disturbance from sensor integration. Shear stresses at the impeller blades ranged from 0.025 to 0.06 Pa, while maximum shear stress ($\tau_{\max}$) values at the vessel base reached approximately 0.1 Pa. Across a range of impeller speeds ($\tau_{\max}$values within the tank were calculated to be 0.043 Pa at Re = 1100 (19 rpm), 0.091 Pa at Re = 2100 (37 rpm), and 0.195 Pa at Re = 5700 (100 rpm), as shown in Figure 2E. These values remain below the critical threshold of 0.25 Pa. Thus, operation with the Bach impeller in water‐like fluids up to 100 rpm does not exceed shear stress levels known to impact cellular function. However, extrapolation of the values indicated that a shear stress of 0.25 Pa, the threshold for potential cell response, would be reached at Re = 6800, corresponding to an impeller speed of 119 rpm. Therefore, while operation at 100 rpm (Re = 5700) remains within a safe range, it approaches the lower limit of shear stress known to affect hMSC behavior. In terms of dissipative length scale, the Kolmogorov length scale estimated from the power number and therefore associated with the average dissipation rate within the tank is found to vary within a range of 113 μm < $\bar{\eta }$ < 190 μm (for 4090 < *Re* < 8180). These values are larger than the typical hMSCs size, 15–30 μm (Krueger et al. 2018), but are comparable to the size of the MCs, ~180 μm, and therefore, viscous stresses at the finest flow scale might affect the process.

**Figure 3:**
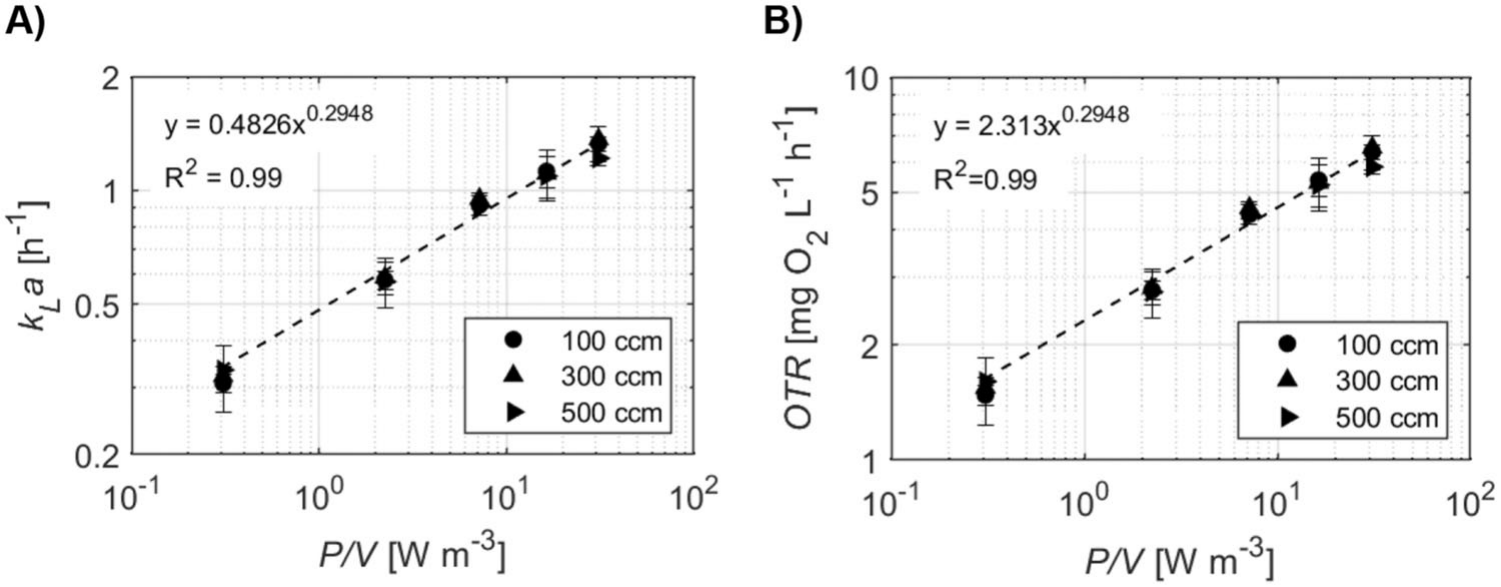
Oxygen transfer characteristics of the Bach impeller with and without submerged probes and lines. (A) Oxygen mass transfer coefficient ($k_{L}a$) and (B) oxygen transfer rate (OTR) for the Bach impeller (D/T = 0.52, C = 0.33 T) with six submerged culture internals (three probes and three lines). Data represents the mean ± SD (*n* = 3). *C*, impeller off‐bottom clearance; *D*, impeller diameter; *T*, vessel tank.

The oxygen transfer capabilities of the bioreactor were then evaluated by measuring the volumetric oxygen mass transfer coefficient ($k_{L}a$) at three air flow rates (100, 300, and 500 cm³/min) introduced into the headspace, across impeller speeds ranging from 50 to 250 rpm. Oxygen transfer from the headspace to the liquid phase increased proportionally with higher impeller speeds and power inputs, as expected. The $k_{L}a$ values exhibited a linear relationship with impeller speed and conformed to a power function, being approximately proportional to ${\left(P/V\right)}^{0.3}$ (Figure 3A). At the lowest impeller speed (50 rpm), $k_{L}a$ was approximately 0.4 h^−1^, increasing to about 1.3 h^−1^ at 250 rpm. These $k_{L}a$ values are comparable to those reported by van Eikenhorst et al. (2014), who found $k_{L}a$ values ranging from 0.3 to 0.8 h^−1^ for P/V between 1 and 15 W/m³ in similar bioreactor configurations.

From the experimentally determined $k_{L}a$ values, the corresponding OTRs were determined (Figure 3B). The ability of these OTRs to support cellular growth depends on the cell‐specific oxygen uptake rate (OUR). Previous studies report that hMSCs derived from adipose tissue consume oxygen at rates of 89 ± 45 × 10^−15^ mol O₂ cell^−1^ h^−1^ (von Heimburg et al. 2005), while bone marrow‐derived hMSCs exhibit OURs of approximately 98–113 × 10^−15^ mol O₂ cell^−1^ h^−1^ (Pattappa et al. 2011). Based on these data, we assumed an OUR of 100 × 10^−15^ mol O₂ cell^−1^ h^−1^ for our WJ‐hMSCs. Table 1 provides a quantitative analysis of how P/V and N relate to the cell densities that can be supported based on the OUR. For instance, to sustain cell densities of 1 × 10⁶ cells/mL, the Bach impeller would theoretically need to operate at approximately 100 rpm (P/V = 2.10 W/m³, $k_{L}a$ ≈ 0.5 h^−1^, OTR = 2.88 mg O₂/h). This agitation speed should be enough to ensure an adequate oxygen supply.

**Table 1:** Estimated supportable cell densities based on the OTR of the Bach impeller at different speeds in a 1‐L bioreactor.

Cell density (cells/mL)	Total cells*	Re (mg O₂/h)	P/V (W/m³)	N (rpm)
2.5 × 10⁵	2.25 × 10⁸	0.72	0.02	19
5 × 10⁵	4.5 × 10⁸	1.44	0.20	43
1 × 10⁶	9 × 10⁸	2.88	2.10	98
1.5 × 10⁶	1.35 × 10⁹	4.32	8.32	158
2 × 10⁶	1.8 × 10⁹	5.76	22.09	222
2.5 × 10⁶	2.25 × 10⁹	7.20	47.08	289

* A final working volume of 900 mL was assumed in the 1 L bioreactor due to samplings over 5–7 culture days. Therefore, the total cell number was calculated as cells/mL × 900 mL.

### 3.3 WJ‐hMSC Culture in 1 L STR

Efficient cell attachment to MCs is essential for establishing viable suspension cultures of WJ‐hMSCs. In this study, WJ‐hMSCs were seeded onto Cytodex 1 MCs at a concentration of 5.6 g/L to evaluate attachment efficiency and subsequent proliferation in a 1 L STR equipped with the Bach impeller. Wyrobnik et al. (2022) indicated that lower rpm values (50–75 rpm) of the Bach impeller provide gentle mixing conditions necessary for WJ‐hMSC culture, while higher speeds (115–150 rpm) can be used to evaluate the effect of increased shear on cell growth and viability. The initial impeller speed was set to 50 rpm for the first 24 h to minimize shear stress and promote cell attachment. After this period, the average attachment efficiency was determined to be 79% ± 5% (*n* = 7). Attachment efficiencies in literature range from 20% to 90% (see review paper from Couto et al. (2020)).

Cultures were maintained at three different impeller speeds: 75, 115, and 150 rpm, to investigate the impact of impeller speed on WJ‐hMSC proliferation and viability. The selection of 75 rpm was informed by prior engineering characterizations, which indicated that this speed ensured the complete suspension of MCs while maintaining a sufficient safety margin to preserve cell integrity. Conversely, 150 rpm was identified as the upper threshold, specifically chosen to assess the effects of elevated shear stress. The intermediate speed of 115 rpm was strategically selected as a midpoint to approximate the arithmetic mean between the lower and upper agitation rates.

**Figure 4:**
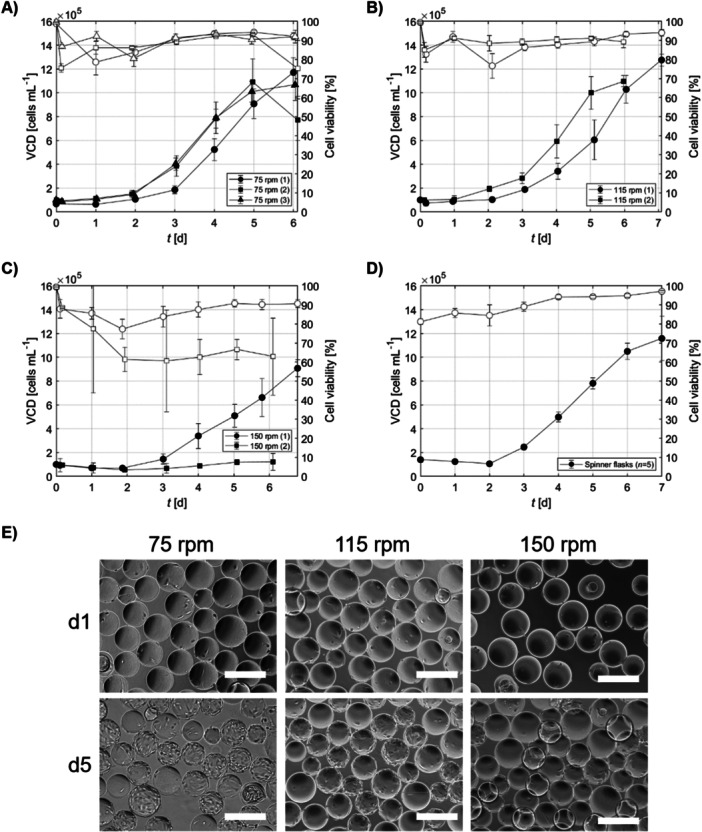
Growth kinetics and cell confluence of WJ‐hMSC bioreactor cultures at various impeller speeds. (A–C) Growth kinetics for WJ‐hMSC cultures in bioreactors operated at 75, 115, and 150 rpm. Each curve represents an independent bioreactor run, denoted by the number in parentheses (e.g., 75 rpm (1), (2), etc.). Black‐filled markers indicate viable cell density (VCD), and white‐filled markers indicate cell viability. (D) Growth kinetics in spinner flasks operated at 30 rpm (*n* = 5 independent runs). Black‐filled markers represent VCD, and white‐filled markers indicate cell viability. (E) Microscopic images of WJ‐hMSCs on Cytodex 1 microcarriers at different impeller speeds. Scale bar = 300 μm. Data represents the mean of technical replicates ± SD (*n* = 3).

**Figure 5:**
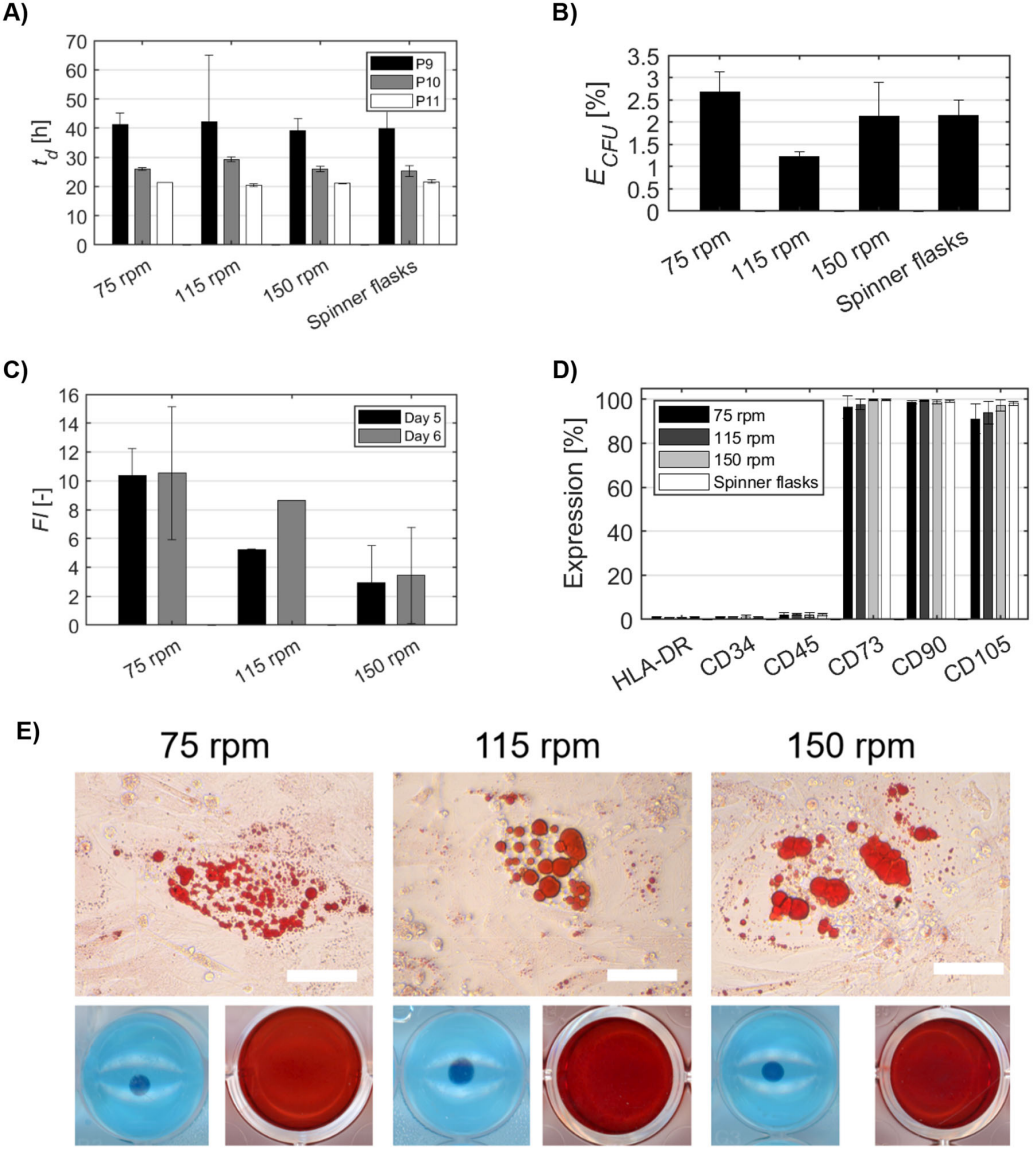
Quality control analyses of WJ‐hMSCs cultured at different impeller speeds. (A) Population doubling time. (B) Colony‐forming unit efficiency across passages. (C) A fold increase in cell numbers at Days 5 and 6. (D) Expression of surface markers. (E) Tri‐lineage differentiation capacity into adipocytes (top, scale bar = 75 μm), osteocytes (bottom left, 24‐well plates), and chondrocytes (bottom right, 96‐well plates). Data represents the mean of technical replicates ± SD (*n* = 3).

The impeller speed was increased from the initial 50 rpm to the designated speeds after the first 24 h of attachment. The growth kinetics over a 7‐day culture period are illustrated in Figure 5. At an impeller speed of 75 rpm, WJ‐hMSCs exhibited exponential growth, achieving cell densities ranging from 1.0 to 1.2 × 10⁶ cells/mL by Days 5 or 6 (Figure 4A). These cell densities are among the highest reported for WJ‐hMSCs in batch cultures using STRs within the 0.5–5 L scale range. For comparison and to the authors' best knowledge, Dosta et al. (2020) documented the highest cell concentrations in MC‐based cultures, achieving 6.05 × 10⁵ cells/mL by Day 7 in a 3 L STR.

The enhanced cell growth at this speed aligns with our oxygen transfer data, indicating that the OTR at 75 rpm met the metabolic demands of the cells, with a $k_{L}a$ close to 0.5 h^−1^. When the impeller speed was increased to 115 rpm, peak cell densities were reached on Days 6 or 7, similar to the growth patterns observed in spinner flask controls operated at 30 rpm (Figure 4B,D). However, at an impeller speed of 150 rpm, WJ‐hMSCs displayed reduced proliferation rates, with one of the cultures failing to grow altogether (Figure 4C). This suggests that the shear stress associated with higher impeller speeds may exceed the tolerance levels of WJ‐hMSCs, potentially causing cellular damage or inducing apoptosis. Microscopic examination confirmed higher cell confluence on MCs at 75 rpm compared to higher impeller speeds (Figure 4E). The population doubling time at 75 rpm was approximately 28 ± 2 h, which is comparable to the 30 ± 3 h observed in spinner flask cultures. In contrast, at an impeller speed of 150 rpm, $t_{d}$ increased to 40 ± 4 h.

**Figure 6:**
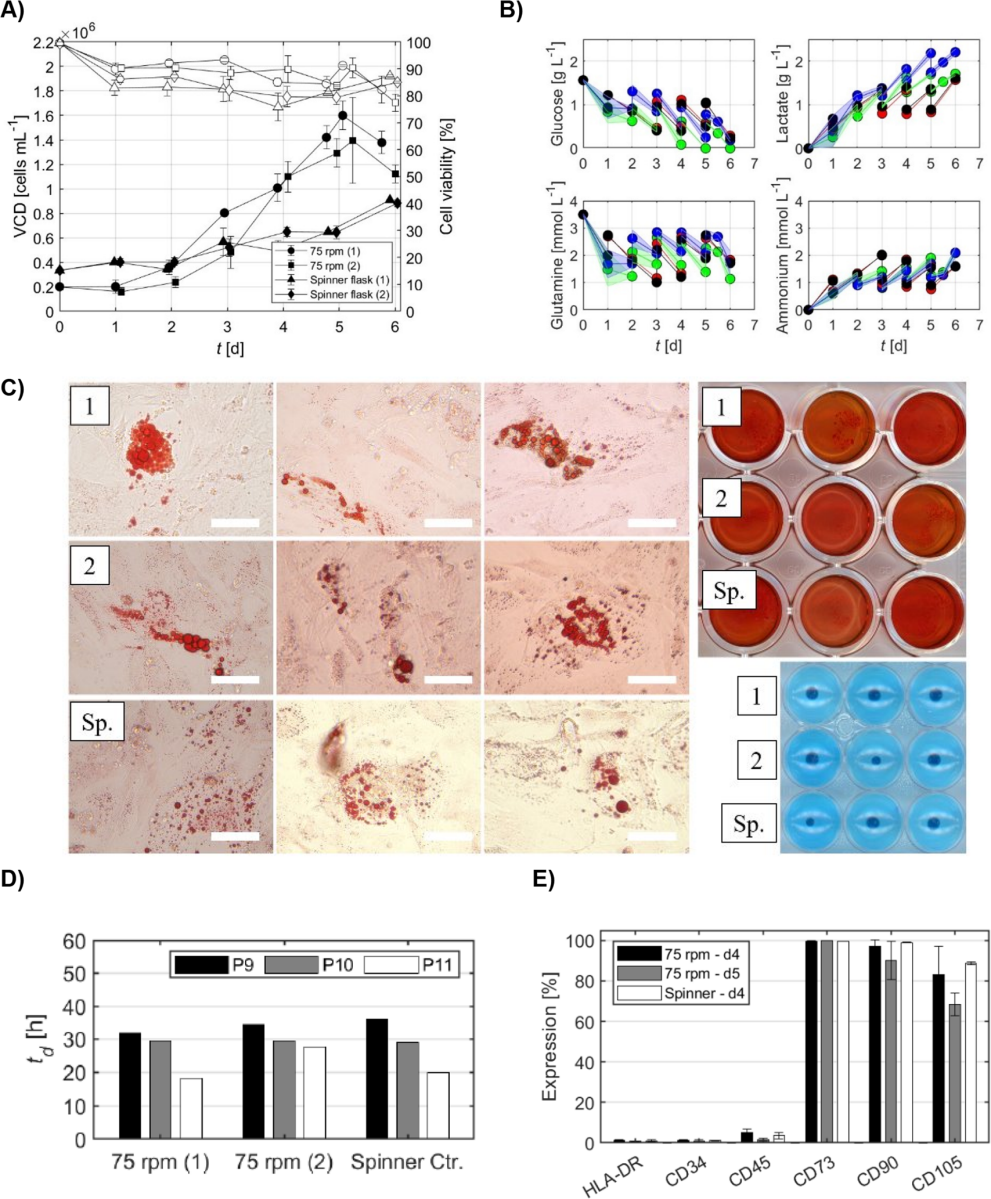
Evaluation of WJ‐hMSC growth and quality metrics at impeller speed of 75 rpm and higher microcarrier concentration (11.2 g/L Cytodex 1). (A) Viable cell density (VCD; black markers) and cell viability (white markers) over the culture period. (B) Metabolite profiles. Data points are color‐coded by condition: green (75 rpm, replicate 1), blue (75 rpm, replicate 2), red (spinner flask control, replicate 1), and black (spinner flask control, replicate 2). (C) Tri‐lineage differentiation capacity of cells harvested from bioreactor and spinner flask in triplicates: adipocytes (left), chondrocytes (top right), and osteocytes (bottom right). Scale bar = 30 μm. (D) Population doubling time for three consecutive passages post‐thawing. (E) Stem cell surface marker expression. Data represents the mean of technical replicates ± SD (*n* = 3).

Quality control analyses demonstrated that the harvested cells cultured at different impeller speeds retained key characteristics. Population doubling times after freeze and thaw remained relatively consistent across the different impeller speeds, with no significant differences observed (Figure 5A). $E_{{\mathrm{CFU}}_{\%}}$ ranged between 1% and 2.5% (Figure 5B). The FI in cell numbers at Days 5 and 6 was highest in cultures operated at 75 and 115 rpm, while the lowest FI was observed at 150 rpm (Figure 5C). Flow cytometry analysis revealed that the expression of key MSC surface markers (CD73, CD90, and CD105) remained consistent across all impeller speeds, with expression levels exceeding 90% (Figure 5D). The markers CD34, CD45, and HLA‐DR remained below 5%, indicating that the impeller speed did not adversely affect the phenotype of the WJ‐hMSCs. Furthermore, the multipotent differentiation potential of WJ‐hMSCs cultured at different impeller speeds was evaluated through tri‐lineage differentiation assays. Cells from all cultures maintained the ability to differentiate into adipogenic, chondrogenic, and osteogenic lineages (Figure 6E), comparable to those grown in spinner flask controls. Overall, while higher impeller speeds were associated with a reduction in cell proliferation, they did not adversely impact the functional and phenotypic properties of WJ‐hMSCs.

Building on the findings from the initial impeller speed investigations at a MC concentration of 5.6 g/L (2× Cytodex 1), we explored increasing the MC concentration to 11.2 g/L (4× Cytodex 1) using the Bach impeller. This increase in MC concentration theoretically provides additional surface area, allowing for a maximum achievable cell density of approximately 2.2 × 10⁶ cells/mL. Preliminary spinner flask trials confirmed the feasibility of reaching cell densities of 2 × 10⁶ cells/mL (Figure S2), establishing a target for the subsequent bioreactor experiments.

Despite the high cell densities achieved at an MC concentration of 5.6 g/L, the process performance at 11.2 g/L was limited by oxygen and nutrient availability. Although peak cell densities of ~1.6 × 10⁶ cells/mL were achieved (Figure 6A), this fell short of the theoretical maximum based on available surface area (∼2.2 × 10⁶ cells/mL). Even with an increase in impeller speed to 115 rpm, the DO levels dropped below the 30% setpoint, indicating that the OTR could not meet the elevated metabolic demands at higher cell densities (Figure S3). Glucose depletion and lactate accumulation further compounded this limitation (Figure 6B). Cells harvested on Day 4 retained the ability to differentiate into the three mesenchymal lineages (Figure 6C), and population times were between 20 and 30 h (Figure 6D). However, substantial reductions in the expression of stem cell surface markers (CD90 and CD105; Figure 6E) were detected on Days 4 and 5. Croughan et al. (1988) and Hewitt et al. (2011) reported deleterious effects associated with approaching or exceeding the maximum cell capacity per MC or increasing MC concentrations (beyond 5.5 g/L). In contrast, Hu et al. (1985) found no decrease in growth rate when bare MCs were added to cultures, or when cultures with 5 g/L MCs were concentrated to 15 g/L. The authors postulated that MC concentration alone does not negatively impact cell growth, provided that initial cell distribution is optimal and adequate nutrient supply is maintained. These findings indicate that oxygen mass transfer may become a limiting factor at elevated MC concentrations. Future investigations should evaluate strategies to enhance oxygen availability, such as employing dual‐impeller configurations or increasing the oxygen partial pressure in the headspace through the use of enriched air or pure oxygen (as opposed to standard atmospheric air at ~21% O₂). Additionally, the implementation of optimized nutrient delivery approaches, such as continuous perfusion, should be considered to support cell growth and viability under these intensified culture conditions in both small‐ and large‐scale bioreactor systems.

Parallel spinner flask controls achieved a maximum cell density of 1 × 10^6^ cells/mL, whereas the bioreactors demonstrated a 40%–60% increase in peak cell density. The difference in cell densities between spinner flask controls and preliminary spinner flask trials remains unexplained. A plausible hypothesis is that the aggregation of cells with MCs may have enhanced survival and proliferation under hypoxic conditions by creating localized microenvironments that supported metabolic adaptation to oxygen limitations. Future studies are needed to quantitatively characterize aggregation behavior and assess its impact on culture performance.

### 3.4 Scaling WJ‐hMSC Culture to 5 L STR

Bioprocess development typically begins in small‐scale devices and is scaled up to larger volumes to conserve resources and improve practicality. However, key process parameters, such as oxygen transfer, mixing, and shear stress, might vary across scales. Successful scale‐up requires retaining the kinetics of the small scale in the larger scale to ensure comparability in cell culture yield or productivity. This is achieved by identifying a critical physical property, the scaling criterion, to be maintained constant across scales. Different criteria can be chosen depending on the most critical physical property for process performance. Common scale‐up criteria include $k_{L}a$, mixing time ($t_{M}$), impeller tip speed ($u_{\mathrm{tip}}$), *N*, flow regime (Re), and P/V (Martin and Vermette 2005; Nienow 2006; Zhang et al. 2010).

A comprehensive analysis compared the effects of scaling up a 1 L process to a 5 L reactor using four scaling criteria (i.e., constant N, Re, P/V, and $u_{\mathrm{tip}}$), for initial conditions of 75 and 115 rpm (see Figure 8, and Wyrobnik (2023) for calculation details). For a 1 L process at 75 rpm, scaling with constant N leads to increased Re, $u_{\mathrm{tip}}$, P/V, and $k_{L}a$ while maintaining $t_{M}$, with these values remaining below inhibitory thresholds observed at 115 rpm; however, scaling with constant P/V would lower the agitation speed to 58 rpm, risking insufficient MC homogenization. In contrast, for a 1 L process at 115 rpm, using $u_{\mathrm{tip}}$ or P/V as the scaling criterion produces more favorable flow conditions, while constant N would generate conditions equivalent to 130–150 rpm in the 1 L system, potentially inhibiting cell growth. Based on these observations and the need for effective MC suspension, the decision was made to scale up using constant impeller rotational speed for both the attachment and growth phases.

**Figure 7:**
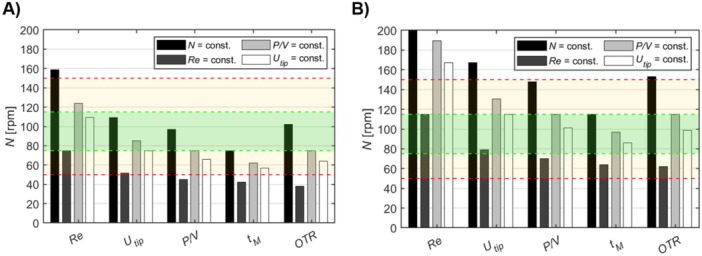
Projected scaling conditions for WJ‐hMSC culture from 1 to 5 L bioreactor. (A) Hypothetical scale‐up of the N = 75 rpm process or (B) a N = 115 rpm process from 1 to 5 L bioreactor, using various scaling criteria. The area highlighted in orange represents the broader design space of rotational speed that may be feasible at the 1 L scale (i.e., 50–150 rpm). The area highlighted in green represents conditions that were shown to reliably offer favorable growth conditions for the WJ‐hMSCs (i.e., 75–115 rpm). Impeller speeds above and below these shaded areas were not viable options for the cell culture. N, agitation speed; *OTR*, oxygen transfer rate; P/V, power input; Re, Reynolds number; *t_M_*, mixing time; $u_{\mathrm{tip}}$, impeller tip speed.

First, scaling up the 1 L process operating at N = 75 rpm by maintaining a constant N results in changes to other key parameters. At N = 75 rpm in the 5 L scale, Re, $u_{\mathrm{tip}}$, P/V, and OTR would all exceed the corresponding values at the 1 L scale, while the $t_{M}$ would remain equivalent. Aside from Re, these parameters would remain below the thresholds previously identified as inhibitory for cell growth at N = 115 rpm (Figure 7A). If the scale‐up is based on maintaining constant P/V, both Re and $u_{\mathrm{tip}}$ would increase relative to the 1 L scale, while $t_{M}$ would be extended, and OTR would be maintained at equivalent levels. While maintaining P/V shows overall promise, N would be reduced to N = 58 rpm. This reduction poses a challenge, as it is insufficient to achieve complete homogenization of MCs throughout the tank volume, as well as ensuring enough oxygen transference (Figure S4).

On the other hand, when scaling up the process at N = 75 rpm using the $u_{\mathrm{tip}}$, the only scaling criteria that solely elevate other critical parameters are constant N or $t_{M}$. In this case, Re at the 5 L scale increases to an equivalent of approximately 115 rpm at the 1 L scale. P/V, both OTR and $t_{M}$ remain similar to those of a 60 rpm process at the 1 L scale. Consequently, concerns regarding MC suspension and oxygen availability rise again. Conversely, for scaling the process at N = 115 rpm, employing $u_{\mathrm{tip}}$ or P/V as the scaling criterion is more advantageous, as these parameters mitigate the relative flow dynamics at the 5 L scale. Maintaining a constant N would produce flow conditions analogous to operating at N = 130–150 rpm at the 1 L scale, which could inhibit cell growth since reliable proliferation was only demonstrated up to N = 115 rpm (green shaded area, Figure 7B).

**Figure 8:**
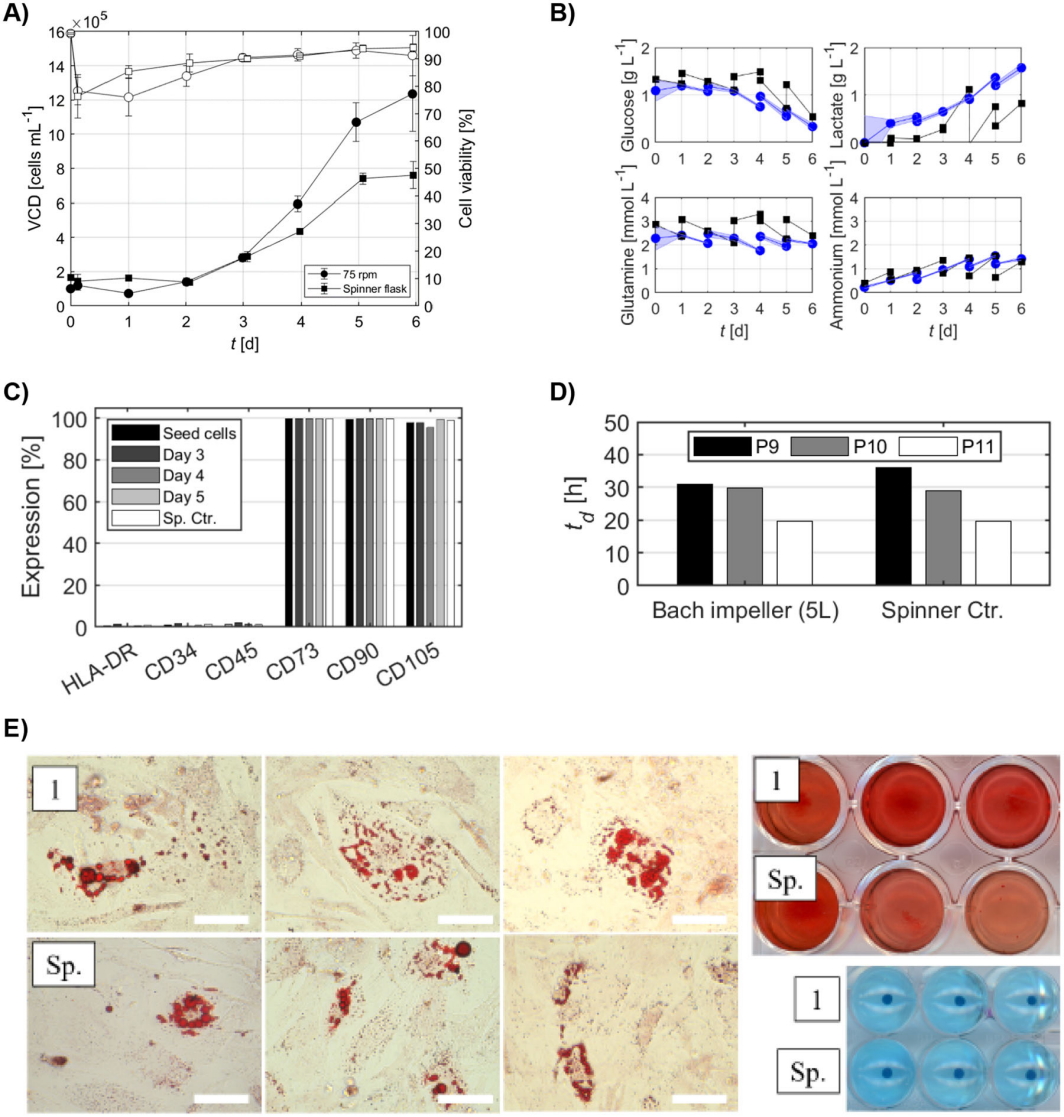
Evaluation of WJ‐hMSC expansion and quality attributes in a 5 L bioreactor. (A) Viable cell density (VCD; black markers) and cell viability (white markers) over the culture period. (B) Metabolite profiles over the culture period. Blue markers indicate samples from the 75 rpm bioreactor condition, and black markers represent those from the spinner flask control. (C) Stem cell surface marker expression. (D) Population doubling time for three consecutive passages post‐thawing. (E) Tri‐lineage differentiation capacity of cells harvested from bioreactor and spinner flask in triplicates: adipocytes (left), chondrocytes (top right), and osteocytes (bottom right). Scale bar = 30 μm. Data represents the mean of technical replicates ± SD (*n* = 3).

Therefore, to evaluate the scalability of the process, we scaled up the WJ‐hMSC culture from 1 to 5 L, maintaining geometric similarity, an impeller speed of 75 rpm, and a MC concentration of 5.6 g/L, based on our earlier findings. As shown in Figure 8A, WJ‐hMSCs in the 5 L STR reached a maximum density of approximately 1.2 × 10⁶ cells/mL within 6 days, comparable to the results obtained at the 1 L scale. Cell viability remained above 90% upon harvest. Metabolite profiles over the culture period were monitored to assess cellular metabolism (Figure 8B). Glucose levels decreased steadily, indicating active consumption, while lactate levels increased, consistent with glycolytic activity. Glutamine consumption and ammonia production were within expected ranges for WJ‐hMSC cultures (dos Santos et al. 2011; Lam et al. 2017). Expression of stem cell markers remained consistent, and WJ‐hMSCs retained the ability to differentiate into adipogenic, osteogenic, and chondrogenic lineages, confirming that scaling up did not compromise cell quality or functionality (Figure 8C). Upon thawing, the cells from the bioreactor displayed equal population doubling times of approximately $t_{d}$ = 20–30 h for P9–P11 compared to the bioreactor cultures at the 1 L scale (Figure 8D). The stemness of the cells was supported by the consistently high expression of stem cell surface markers such as CD73 (> 99%), CD90 (> 99%), and CD105 (> 95%), and absence of negative markers CD34, CD45, and HLA‐DR (all < 2%) on cells from Days 3, 4, and 5 post‐seeding (Figure 8E).

## 4 Discussion

This study presents a comprehensive evaluation of the Bach impeller for the scalable expansion of WJ‐hMSCs on MCs in STRs. Our findings demonstrate that the Bach impeller provides a favorable hydrodynamic environment for WJ‐hMSC culture, enabling high cell densities while maintaining cell viability and functionality. The use of the Bach impeller at 75 rpm in a 1 L STR resulted in a maximum cell density of approximately 1.2 × 10^6^ cells/mL within less than a week, surpassing many previously reported values for WJ‐hMSC batch cultures in STRs. In fact, we obtained 1.6 × 10^6^ cells/mL when doubling the concentration of MCs. Rafiq et al. (2013) achieved cell densities of around 5 × 10^5^ cells/mL using a 3BS impeller in a 5 L STR, while Lam et al. (2017) reported densities up to 8 × 10^5^ cells/mL with a pitched‐blade impeller. Yuan et al. (2018) used vertical‐wheel bioreactors and achieved cell densities up to 1 × 10^6^ cells/mL. Schirmaier et al. (2014) reported cell densities of approximately 8 × 10^5^ cells/mL using conventional impellers but noted increased cell damage at higher speeds. To the authors' knowledge, previous studies in STRs reach around 10^5^ cells/mL. This corresponds to a 2‐ to 10‐FI in cell density achieved in a shorter cultivation time, depending on the initial conditions. These findings suggest that previous assumptions regarding required bioreactor volumes could potentially be reevaluated. For instance, if cell densities around 10^6^ cells/mL can be maintained, this could increase productivity and reduce the necessary bioreactor volume by an order of magnitude, though further investigation would be needed to confirm this at scale. The higher cell densities observed in our study can be attributed to the optimized mixing and low shear environment provided by the Bach impeller.

Our results indicate that impeller speed significantly influences WJ‐hMSC proliferation and viability. At 75 rpm, cells exhibited exponential growth and high viability (> 90%). However, increasing the speed to 150 rpm led to reduced growth rates and, in some cases, culture failure. This aligns with the findings of Schirmaier et al. (2014), who reported that hMSCs are sensitive to shear stress, with optimal growth occurring at lower impeller speeds that minimize turbulent energy dissipation rates. Importantly, cells expanded using the Bach impeller retained their critical quality attributes. Flow cytometry analyses showed high expression of mesenchymal markers (CD73, CD90, CD105) and low expression of hematopoietic markers (CD34, CD45, HLA‐DR), consistent with the criteria established by the International Society for Cellular Therapy (Dominici et al. 2006). Additionally, the cells preserved their differentiation potential into adipogenic, osteogenic, and chondrogenic lineages, demonstrating that the culture conditions did not induce unwanted phenotypic changes. It should be noted, however, that biological evaluation in this study focused primarily on cell growth and identity; detailed functional assessments were beyond the scope of this study.

Scaling up the process to a 5 L STR while maintaining an impeller speed of 75 rpm resulted in comparable cell densities and quality attributes. This suggests that the hydrodynamic conditions optimized at the small scale can be effectively translated to larger volumes, a critical factor for industrial applications. Previous studies have often encountered challenges during scale‐up due to changes in mixing and shear profiles (Nienow 2006; Nienow et al. 2016). Padhiar et al. (2022) cultured WJ‐hMSCs in a 5 L STR using MCs, reaching 5 × 10^5^ cells/mL in 14 days. Tozetti et al. (2017) expanded umbilical cord‐derived MSC cultures in a 2 L bioreactor, reaching 1 × 10^5^ cells/mL by Day 7. Large‐scale studies (Lawson et al. 2017; Schirmaier et al. 2014) have achieved up to 2.56 × 10^5^ cells/mL and 2.8 × 10^5^ cells/mL, respectively, in 50 L bioreactors. More recently, Chen et al. (2021) used bead‐to‐bead cell transfer to scale up bone marrow‐derived MSC cultures from 4 to 50 L. By using P/V as the scaling parameter, they reached a final cell density of 2.9 × 10^5^ cells/mL after 27 days. In contrast, our method not only achieves higher cell densities (1 × 10^6^ cells/mL vs. 1 × 10^5^ cells/mL) in significantly shorter times (5–6 days vs. up to 30 days) but also implies that industrial‐scale production could be more efficient and cost‐effective. For context, to achieve the same final cell number than Chen et al. (2021) at a concentration of 1 × 10^6^ cells/mL, one would require a 15 L reactor instead of a 50 L. These comparisons should be interpreted with caution, as cell source and doubling times vary across studies.

The substantial increase in cell density and the reduction in cultivation time achieved in our study suggest that bioreactor sizes could potentially be reduced by up to 90%. This would result in substantial reductions in both spatial and operational requirements. While scale‐up in this study was guided by engineering characterization and successfully executed at 5 L by maintaining constant impeller speed, future investigations should include comparisons across these common scale‐up criteria to determine which most effectively preserves the optimal hydrodynamic conditions and biological outcomes when transitioning from small‐scale (e.g., 1 and 5 L) to larger bioreactor volumes (≥ 20 L). Furthermore, recent advances in hMSC bioprocessing have demonstrated the feasibility of transitioning from adherent to suspension culture. Silva Couto et al. (2024) reported that suspension‐adapted human mesenchymal stromal cells can retain their viability and functional properties. This shift presents a promising avenue to further enhance manufacturing efficiency, as suspension‐based systems eliminate the need for MCs, simplify downstream processing, and are inherently more scalable. Collectively, the engineering insights from our study, combined with emerging suspension‐adaptation strategies, suggest a compelling new direction for hMSC manufacturing.

## 5 Conclusions

In summary, the Bach impeller enabled the expansion of WJ‐hMSCs on MCs to a density of up to 1.6 × 10^6^ cells/mL in just 5 culture days, exceeding previously reported values and demonstrating that this approach is well‐suited for high‐density WJ‐hMSC cultivation. Critical cell quality attributes, including viability (> 90%), surface marker expression, and tri‐lineage differentiation potential, were maintained, even upon scaling the process from 1 to 5 L STR. These results highlight the scalability and robustness of this system, indicating that further increases to larger‐scale manufacturing are likely achievable while preserving both high cell yield and therapeutic quality.

## Nomenclature

### Symbols

$A_{\%}$
: percentage of attached cells, %
C
: impeller off‐bottom clearance, mm
$C_{\mathrm{DO}}$
: dissolved oxygen concentration in liquid, mg/L
$C^{*}$
: saturated oxygen concentration in liquid, mg/L
$C_{x}$
: viable cell count before harvesting, cells/mL
$C_{x}^{*}$
: viable cell count after harvesting, cells/mL
${\text{Cx}}_{0}$
: initial cell count, cells/mL
${\text{Cx}}_{\mathrm{susp}}$
: viable cell number in suspension, cells/mL
${\text{Cx}}_{t}$
: final cell count, cells/mL
${\text{Cx}}_{\mathrm{tot}}$
: total viable cell number, cells/mL
D
: impeller diameter, mm
$E_{{\mathrm{CFU}}_{\%}}$
: colony‐forming unit efficiency, %
FI
: fold increase, %
H
: tank height, mm
$H_{\%}$
: harvesting efficiency, %
$k_{L}a$
: volumetric oxygen mass transfer coefficient, h^−1^
μ
: specific growth rate, h^−1^
n: number of replicates
N
: impeller speed, revolutions per minute (rpm)
$N_{P}$
: power number, −
OTR
: oxygen transfer rate, mg O₂/L·h
P
: power input, W
P/V
: volumetric power input, W/m³
$Q_{\mathrm{air}}$
: air flow rate, cm^3^/min
Re
: Reynolds number, −
$t_{d}$
: population doubling time, h
$t_{M}$
: mixing time, s
T
: tank diameter, mm
$u_{\mathrm{tip}}$
: impeller tip speed, m/s
$V_{W}$
: working volume, L
Δt
: time interval between passages, h

## Author Contributions

**Tom A. Wyrobnik:** conceptualization, methodology, formal analysis, investigation, data curation, writing – original draft, writing – review and editing, visualization. **Laia Miranda:** data curation, writing – original draft, writing – review and editing, visualization. **Alan Lam:** conceptualization, writing – review and editing, supervision, project administration. **Steve Oh:** conceptualization, writing – review and editing, supervision, project administration, funding acquisition. **Andrea Ducci:** conceptualization, methodology, formal analysis, resources, writing – review and editing, visualization, supervision. **Martina Micheletti:** conceptualization, methodology, writing – review and editing, visualization, supervision, project administration, funding acquisition.

## Conflicts of Interest

The authors declare no conflicts of interest.

## Supporting information

hMSC SupplMaterial.

## Data Availability

The data that support the findings of this study are available from the corresponding author upon reasonable request.
