# Supplementary material for: Scalable, High‐Density Expansion of Human Mesenchymal Stem Cells on Microcarriers Using the Bach Impeller in Stirred‐Tank Reactors
Source: Biotechnol Bioeng. 2025 Jul 17;122(10):2803–18. doi: 10.1002/bit.70025 (PMC12417783; doi:10.1002/bit.70025)
Supplement: Supplementary file 1 — hMSC SupplMaterial. [file BIT-122-2803-s001.docx]

**Supplementary material**

| **A)** | **B)** | **C)** |
| --- | --- | --- |
| 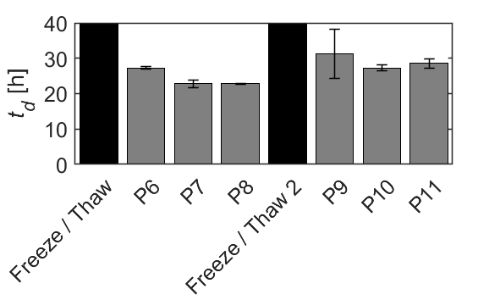 | 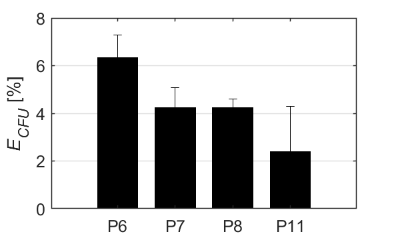 | 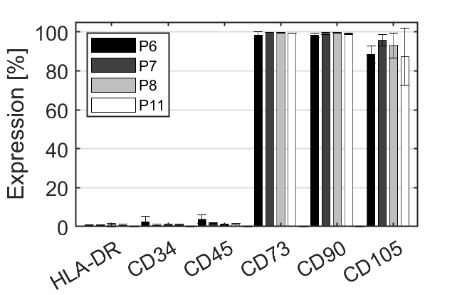 |
| **D)** | **E)** | **F)** |
| 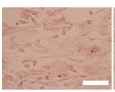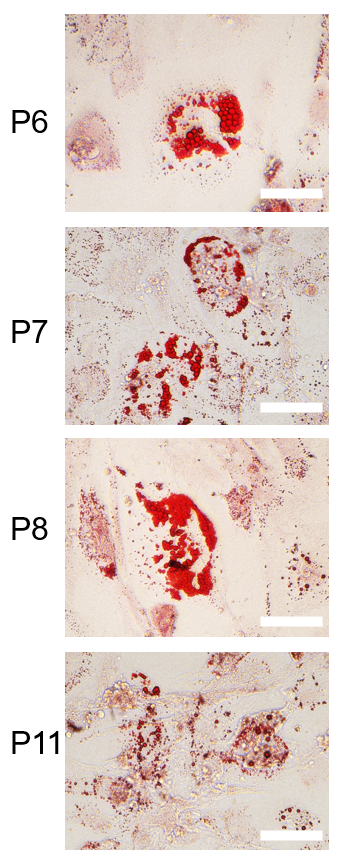  Ctr. | 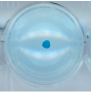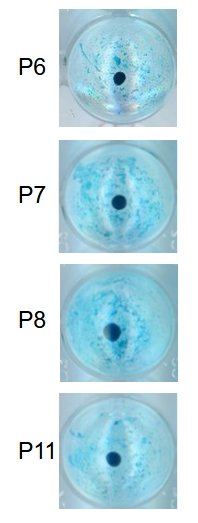  Ctr. | 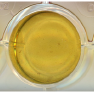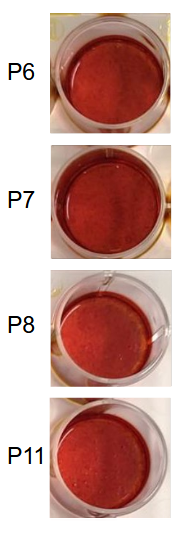  Ctr. |

***Supplementary Figure 1:*** ***Baseline performance metrics for WJ-hMSCs cultured on planar surfaces.*** *(A) Population doubling time at different passages. (B) Colony-forming unit efficiency across passages. (C) Expression of stem cell surface markers. (D-F) Tri-lineage differentiation assay (D) Adipogenic differentiation (scale bar = 75 μm). (E) Chondrogenic differentiation (24-well plate). (F) Osteogenic differentiation (96-well plate). Data represents mean of technical replicate ± SD (n = 3).*

| A) |
| --- |
| 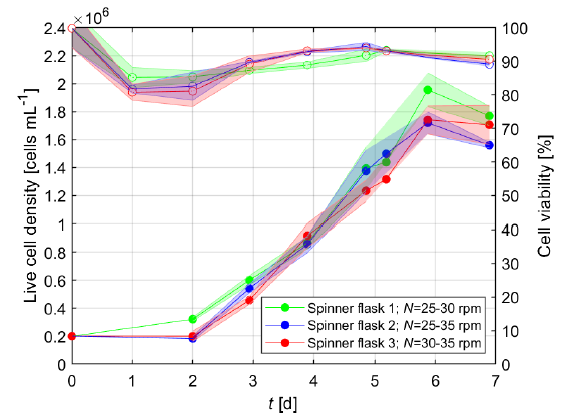 |
| B) |
| 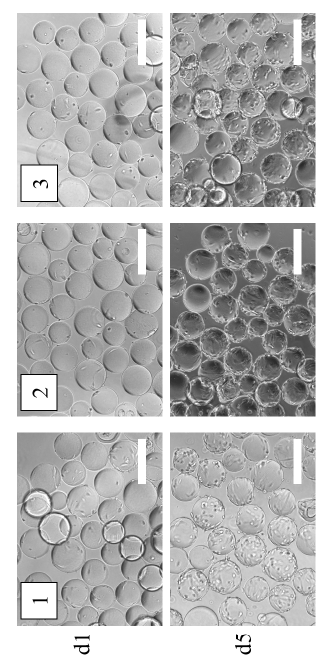 |

***Supplementary Figure 2****:* ***Growth kinetics, cell viability, and confluence in spinner flasks at high microcarrier concentration (4X = 11.2 g/L Cytodex 1).*** *(A) Growth kinetics in spinner flasks. Full symbols correspond to the live cell density while empty symbols correspond to the cell viability. (B) Microscope images of 3 spinner flasks from 4X trial. Image column 1:* $N$ *= 25-30 rpm; column 2:* $N$ *= 25-35 rpm; column 3:* $N$*= 30-35 rpm. Scale bar = 300 μm. Data is shown as mean ± SD (n = 3).*

| A) |
| --- |
| 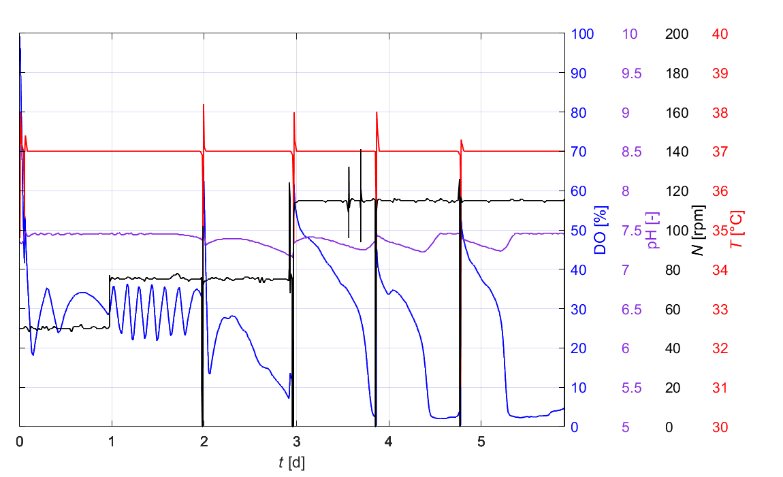 |
| B) |
| 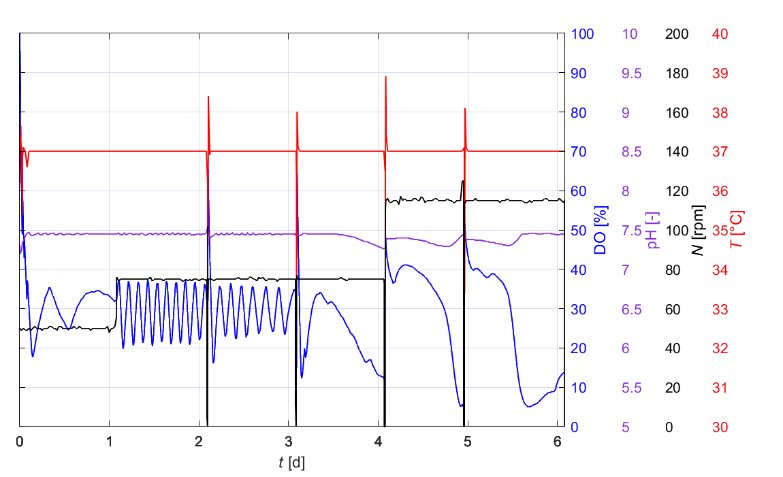 |

***Supplementary Figure 3****:* ***Bioreactor parameters for the two parallel bioreactor runs (A-B) at Cytodex 11.2g/L and*** $\boldsymbol{N}$***= 50-75 rpm with the Bach impeller****. For all tested conditions, the dissolved oxygen (DO), temperature (T), and pH were set to 30%, 37°C, and 7.4, respectively. Working volume (*$V_{W}$*) = 1 L.*

| A) | B) | C) |
| --- | --- | --- |
| 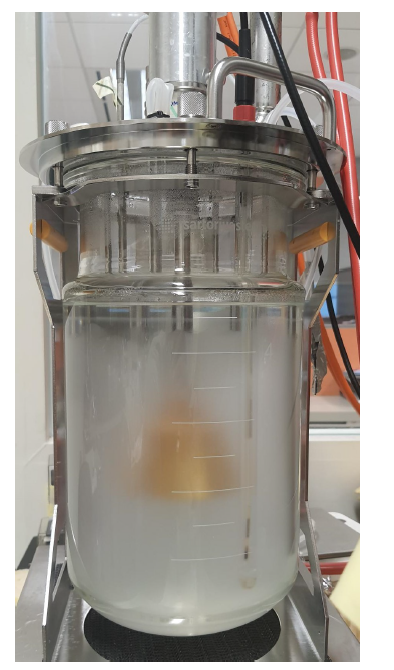 | 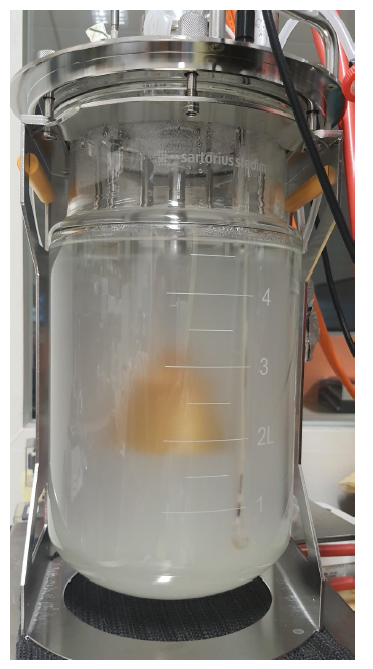 | 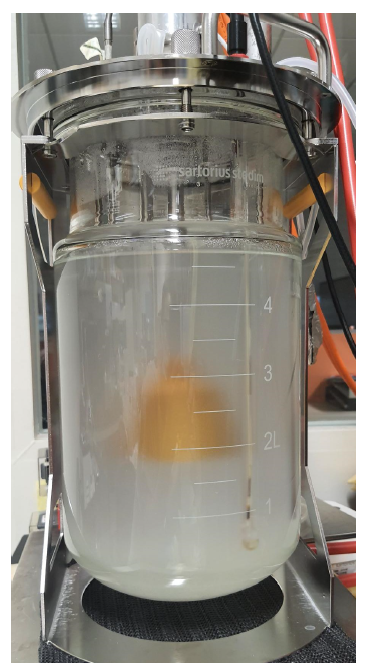 |

***Supplementary Figure 4****:* ***Suspension of Cytodex 1 microcarriers (5.6 g/L) in PBS under varying agitation conditions.*** *The system consists of a Bach impeller (*$D/T$ *= 0.52,* $C$ *= 0.6*$T$*) in a 5 L vessel at (a) 50 rpm, (b) 60 rpm, and (c) 70 rpm.* $D$*: impeller diameter,* $T$*: vessel tank.*
